# Quantifying the Relationship Between Renal Function and Procalcitonin: A Study of 14 431 Blood Cultures

**DOI:** 10.1093/ofid/ofaf654

**Published:** 2025-10-21

**Authors:** Albert K Park, Trisha S Nakasone, Amit Kaushal, Cybèle Renault

**Affiliations:** Department of Medicine, Stanford University School of Medicine, Stanford, California, USA; Veterans Affairs Palo Alto Health Care System, Palo Alto, California, USA; Department of Medicine, Stanford University School of Medicine, Stanford, California, USA; Veterans Affairs Palo Alto Health Care System, Palo Alto, California, USA; Department of Medicine, Stanford University School of Medicine, Stanford, California, USA; Veterans Affairs Palo Alto Health Care System, Palo Alto, California, USA; Division of Infectious Diseases and Geographic Medicine, Stanford University Medical Center, Stanford, California, USA

## Abstract

**Background:**

Negative procalcitonin (PCT) values may decrease suspicion for bacterial bloodstream infections among patients presenting with symptoms of infection; however, the reliability of PCT in patients with renal dysfunction remains poorly characterized. We quantified the relationship between estimated glomerular filtration rate (eGFR) and positive PCT values in patients with suspected bacterial bloodstream infections.

**Methods:**

We conducted a retrospective cohort study of 2,832 patients with 14 431 blood cultures at a Veterans Affairs Medical Center (2016–2024). PCT values within 48 hours of culture were analyzed across eGFR categories based on chronic kidney disease staging. We used multivariate logistic regression to assess the relationship between eGFR and positive PCT (≥0.5 ng/mL), adjusting for age and race.

**Results:**

Among 14 431 blood cultures, 1417 (9.8%) were positive. When cultures were positive, the proportion of positive PCT values was 47.0% with normal renal function (eGFR ≥90 mL/min/1.73m²) and 91.7% in severe renal dysfunction (eGFR 0–14.9 mL/min/1.73m²). When cultures were negative, the proportions of positive PCT values were 27.6% and 84.0%, respectively. For each lower eGFR category, the odds of a positive PCT value were 40–43% higher (OR 1.40 for positive cultures, 1.43 for negative cultures), after adjusting for age and race. This inverse relationship was consistent across multiple PCT thresholds (0.25–1.50 ng/mL).

**Conclusions:**

This large-scale analysis uncovers an inverse relationship between renal function and positive PCT values in patients both with and without culture-positive bloodstream infections. The discriminatory power of PCT for bloodstream infection diminishes in patients with impaired renal function.

## Teaser

We observed an inverse relationship between renal function and procalcitonin positivity. Each successive category of worsening renal function increases the odds of positive procalcitonin values by approximately 40%–43%.

Bloodstream infections remain a leading cause of morbidity and mortality worldwide [1]. Alarming rates of antimicrobial resistance have led to efforts to reduce inappropriate antimicrobial use. In this regard, reliable diagnostic biomarkers predicting bloodstream infection can support antimicrobial stewardship efforts. Procalcitonin (PCT), a precursor peptide to calcitonin, is often significantly elevated in the setting of bacterial infections, including bloodstream infections [2].

From an antimicrobial stewardship perspective, PCT has been most rigorously evaluated for reducing antimicrobial duration in clinically stable, immunocompetent patients hospitalized with suspected bacterial pneumonia [3–5]. While the typical duration for treatment of community-acquired pneumonia is 5 days in clinically improving patients [6], PCT trends can help guide decisions when extending therapy for patients with delayed improvement. Early European randomized controlled trials (RCTs) established that PCT-guided algorithms for antibiotic management in patients with lower respiratory tract infections (LRTIs) reduced antibiotic exposure while maintaining patient safety [4, 7]. Additionally, persistently low PCT values on serial measurements can support discontinuation of empiric antibiotics, particularly when alternative diagnoses such as viral pneumonia [8], pulmonary edema [9], acute bronchitis or COPD exacerbation are suspected [4].

As a diagnostic marker, PCT demonstrates sensitivities of 66–89% in critically ill patients with sepsis and suspected bloodstream infections [10]. A meta-analysis of 11 RCTs studying the utility of PCT-guided antimicrobial discontinuation in this patient group demonstrated that PCT-guided decision-making reduces both antibiotic duration (9.3 vs 10.4 days, *P* < .001) and inpatient mortality (21.1% vs 23.7%, *P* = .03) [11]. In 2016, a randomized open-label trial demonstrated a mortality benefit with PCT-guided antibiotic discontinuation in this population [12]. Although subsequent studies and meta-analyses have questioned the mortality benefit observed in these two studies, they consistently show that PCT-guided therapy achieves shorter antibiotic durations, a core goal of antimicrobial stewardship [13–15]. Although its clinical use has not been standardized, PCT has been investigated across various infection types [16–19] and clinical settings [20–22], with its incorporation into clinical decision-making algorithms expanding across US hospitals [22–24].

PCT reliability may be affected in patients with renal insufficiency, though the clinical implications of this relationship remain to be fully established [25]. Two potential mechanisms explain this concern: PCT's primary clearance occurring through the kidneys [26–28] and the chronic inflammatory state characteristic of patients with dialysis-dependent chronic kidney disease (CKD) [29–31]. Studies have demonstrated reduced sensitivity and specificity of PCT in patients with severe renal impairment and suspected bloodstream infections [32–34], though these investigations were limited by small sample sizes. A meta-analysis of 803 patients found that PCT cutoffs ranging from 0.8–2.0 ng/mL improved diagnostic performance compared with the traditional 0.5 ng/mL threshold in patients with renal insufficiency [35]. Given that CKD affects approximately 14% of the US population [36, 37], an improved understanding of the relationship between renal function and PCT interpretation is critical for clinical decision-making in patients with suspected bloodstream infections.

Despite the evidence that renal function impacts PCT values in bloodstream infection diagnosis, no large-scale studies have quantified this relationship in a well-characterized cohort. In this work, we conducted an observational cohort study of 2832 patients with 14 431 blood cultures to validate and quantify the inverse relationship between estimated glomerular filtration rate (eGFR) and PCT in suspected bloodstream infection. Using multivariate regression analyses and comprehensive interaction modeling, we examined the association between varying degrees of renal dysfunction and positive PCT values in both culture-positive and culture-negative cases, while accounting for demographic factors and testing four PCT positivity thresholds. To our knowledge, this represents the first large-scale investigation to quantify the relationship between concurrent PCT and eGFR measurements in patients with suspected bloodstream infections.

## METHODS

### Study Design and Population

This retrospective observational study was conducted using data from the Veterans Affairs Palo Alto Health Care System, extracted from PraediAlert™ clinical surveillance software following Institutional Review Board approval. We analyzed data from adult patients (age ≥18 years) with both PCT measurements and blood cultures drawn between 1 January 2016 (when PCT testing was initiated at our institution) and 30 August 2024. The study included both inpatient and outpatient settings (intensive care units, hospital wards, emergency department, and outpatient clinics). Data collection included patient demographics (age, race), laboratory test results including PCT and eGFR (as calculated by the VA laboratory system), and blood culture results. Patients could contribute multiple episodes of data during the study period.

### Data Processing and Variable Definitions

We performed initial data processing using Python (version 3.12) with pandas, numpy, scipy.stats, and statsmodels packages. Blood cultures were classified as positive or negative based on whether a bacterial species was identified. Each culture bottle was treated as an independent unit of analysis. Polymicrobial cultures (defined as two or more bacterial species identified within 24 hours of initial culture collection) were included, with each species counted as a separate positive culture, representing 310 cultures across 78 episodes (21.9% of positive cultures). For each blood culture, we identified the PCT value measured closest to culture collection within a 48-hour window to capture PCT values that would be clinically relevant to the infection episode while maintaining temporal proximity to culture collection. We excluded the following: (1) positive blood cultures without bacterial species identification, (2) negative blood cultures drawn within 24 hours of a positive culture from the same patient to avoid misclassification of culture sets where any bottle was positive, and (3) blood cultures where the identified PCT value did not have a corresponding eGFR measurement from the same calendar day. PCT values ≥0.5 ng/mL were considered positive based on established literature [38].

### Statistical Analysis

Statistical analyses were performed using R software (version 4.4.2). Baseline characteristics were summarized using means with standard deviations for continuous variables and frequencies with percentages for categorical variables. For all analyses involving individual cultures, age was calculated at the time of culture collection.

We categorized eGFR values (as calculated by the VA laboratory system using the 2021 CKD-EPI equation) into clinically relevant categories aligned with CKD staging: 0–14.9, 15–29.9, 30–44.9, 45–59.9, 60–74.9, 75–89.9, and ≥90 mL/min/1.73m² [39]. Multiple cultures from the same patient were treated as independent episodes.

We performed multivariate logistic regression analyses using positive PCT (≥0.5 vs <0.5 ng/mL) as the binary outcome and eGFR category (ordinal, 1–7) as the primary predictor, adjusting for age (continuous) and race (categorical: White [reference], Asian, Black/African American, Hispanic/Latino, Pacific Islander/Native Hawaiian, and Other). Sex was excluded from adjustment due to the predominance of male patients (96.3%). Separate models were fitted for positive and negative blood cultures.

To assess whether the relationship between eGFR and PCT positivity differed by culture status, we constructed an interaction model combining both positive and negative culture data. This model included an interaction term between eGFR category and culture status (positive/negative), while maintaining the same covariates (age and race) as the primary analysis.

We conducted sensitivity analyses using four PCT positivity thresholds (0.25, 0.50, 1.00, and 1.50 ng/mL) to assess whether our findings were consistent across PCT positivity thresholds. For each threshold, we calculated adjusted odds ratios with 95% confidence intervals and corresponding *P*-values to assess the robustness of the eGFR-PCT relationship across different PCT cutoff values.

## RESULTS

### Patient and Culture Characteristics

Among 2832 patients analyzed, the mean age was 76.7 ± 12.7 years. The cohort was predominantly male (96.3%) and White (70.1%), with an average of 5.1 ± 5.3 blood cultures per patient (Table 1A). Of 14 431 total blood cultures, 1417 (9.8%) were positive and 13 014 (90.2%) were negative. Most cultures were collected in acute care settings: emergency department (46.4%), intensive care unit (34.2%), and inpatient wards (18.2%), with the remaining 1.2% collected in outpatient clinics and other settings. The proportion of positive cultures remained stable across collection locations, ranging from 8.7% to 10.4%. The proportion of positive cultures showed minimal variation between the early (2016–2020: 9.2%) and late (2021–2024: 10.4%) study periods (Table 1B).

**Table 1A.** Baseline Patient Demographics.

Patient Characteristics (n = 2832)	Value
Age^a^, years: mean ± SD	76.7 ± 12.7
Sex: n (%)	…
Male	2,727 (96.3%)
Female	105 (3.7%)
Race: n (%)	…
White	1986 (70.1%)
Black/African American	296 (10.5%)
Asian	119 (4.2%)
Pacific Islander/Native Hawaiian	93 (3.3%)
Hispanic/Latino	81 (2.9%)
Other	257 (9.1%)
Blood cultures per patient: mean ± SD	5.1 ± 5.3

^a^Age calculated as of 30 August 2024, regardless of mortality status.

**Table 1B.** Blood Culture Characteristics.

	Positive Cultures (n = 1417)	Negative Cultures (n = 13 014)	Total (n = 14 431)
**Time Period: n (% of total)**	…	…	…
2016–2020	665 (9.2%)	6528 (90.8%)	7193
2021–2024	752 (10.4%)	6486 (89.6%)	7238
**Collection Location: n (% of total)**	…	…	…
Emergency Department	699 (10.4%)	6001 (89.6%)	6700
ICU	428 (8.7%)	4505 (91.3%)	4933
Ward	273 (10.4%)	2349 (89.6%)	2622
Outpatient	5 (9.6%)	47 (90.4%)	52
Other	12 (9.7%)	112 (90.3%)	124
**eGFR Value (mL/min/1.73m²): n (% of total)**	…	…	…
≥90	347 (8.9%)	3573 (91.1%)	3920
75–89.9	144 (8.1%)	1643 (91.9%)	1787
60–74.9	174 (7.9%)	2023 (92.1%)	2197
45–59.9	203 (9.8%)	1866 (90.2%)	2069
30–44.9	241 (12.2%)	1733 (87.8%)	1974
15–29.9	187 (11.5%)	1434 (88.5%)	1621
0–14.9	121 (14.0%)	742 (86.0%)	863
**PCT Value (ng/mL): n (% of total)**	…	…	…
<0.5	505 (6.3%)	7519 (93.7%)	8024
≥0.5	912 (14.2%)	5495 (85.8%)	6407

SD, standard deviation; ICU, intensive care unit; PCT, procalcitonin; eGFR, estimated glomerular filtration rate.

### Proportion of Positive PCT Values Across eGFR Categories

**Figure 1.**
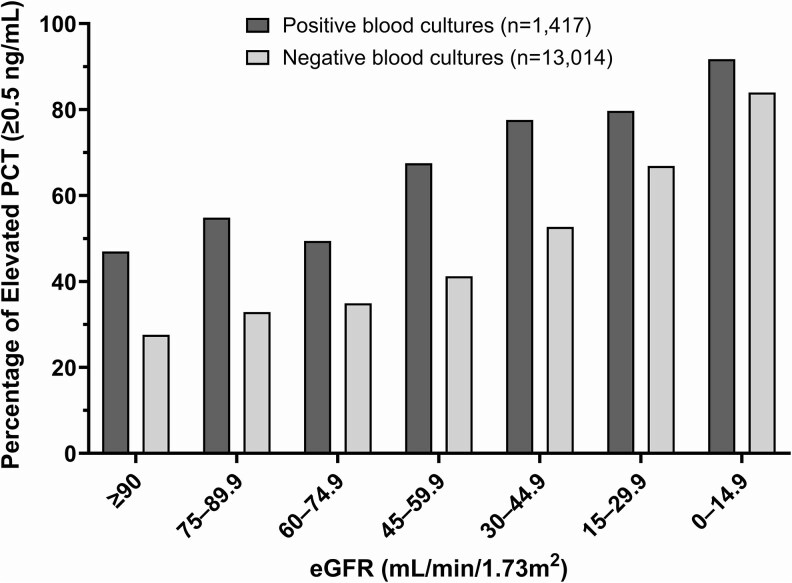
Percentage of positive procalcitonin values stratified by eGFR categories in positive and negative blood cultures. Bar graphs show the percentage of PCT values ≥0.5 ng/mL across eGFR categories for positive (dark gray, n = 1417) and negative (light gray, n = 13 014) blood cultures. PCT, procalcitonin; eGFR, estimated glomerular filtration rate.

We observed an increase in the proportion of positive PCT values (≥0.5 ng/mL) as renal function decreased. With positive cultures, the proportion of positive PCT values ranged from 47.0% in patients with normal renal function (eGFR ≥90 mL/min/1.73m²) to 91.7% in those with severe renal dysfunction (eGFR 0–14.9 mL/min/1.73m²). A similar pattern emerged in the setting of negative cultures, with the proportion of positive PCT values increasing from 27.6% to 84.0% across the same eGFR spectrum (Figure 1, Table 2).

**Table 2.** EGFR-stratified Procalcitonin Levels in Positive and Negative Blood Cultures.

…	Positive Blood Cultures (n = 1417)	Negative Blood Cultures (n = 13 014)
**Percentage of Positive PCT (≥0.5 ng/mL) by eGFR Categories (%)**	…	…
≥90	47.0	27.6
75–89.9	54.9	32.9
60–74.9	49.4	34.9
45–59.9	67.5	41.2
30–44.9	77.6	52.7
15–29.9	79.7	66.9
0–14.9	91.7	84.0
**Adjusted** ^a^ **Odds Ratio of Positive PCT for Each Lower EGFR Category**	1.40	1.43
95% Confidence Interval	1.32–1.49	1.41–1.45
*P*-Value	<0.001	<0.001

^a^Adjusted for age and race.

PCT, procalcitonin; eGFR, estimated glomerular filtration rate.

#### Multivariate and Interaction Analyses

In multivariate analyses adjusted for age and race (full analysis in Supplementary Table 1), lower eGFR categories were associated with significantly higher odds of positive PCT values in both positive (adjusted OR = 1.40, 95% CI: 1.32–1.49, *P* < .001) and negative (adjusted OR = 1.43, 95% CI: 1.40–1.46, *P* < .001) blood cultures (Table 2). The analysis also revealed that, compared with White patients, Asian patients with positive blood cultures (adjusted OR = 3.36, 95% CI: 1.58–8.33, *P* = .004) and Black/African American patients with negative blood cultures (adjusted OR = 1.44, 95% CI: 1.28–1.62, *P* < .001) had higher odds of PCT positivity, although these groups comprised only 4.2% and 10.5% of the study population, respectively.

To examine whether the eGFR-PCT relationship differed by culture status, we constructed an interaction model combining all 14 431 cultures (Supplementary Table 2). The model revealed associations after adjustment between PCT positivity and both eGFR (β = −0.357, 95% CI: −0.377 to −.337, *P* < .001) and culture positivity (β = 0.897, 95% CI: .571 to 1.223, *P* < .001). The interaction term between eGFR and culture status was not significant (β = −0.001, 95% CI: −.065 to .062, *P* = .966), indicating that the relationship between eGFR and PCT positivity remained consistent regardless of the culture result. Increasing age was associated with lower odds of PCT positivity (β = −0.012, 95% CI: −.015 to −.009, *P* < .001). Compared to White race, Asian (β = 0.213, 95% CI: .032 to .395, *P* = .021) and Black/African American (β = 0.363, 95% CI: .250 to .476, *P* < .001) classifications showed higher odds of PCT positivity, while other racial categories showed no significant associations.

### Sensitivity Analysis

The inverse relationship between eGFR and PCT positivity was consistent across the four PCT positivity thresholds. Adjusted odds ratios for PCT positivity with each eGFR category decrease ranged from 1.25 to 1.57 for positive cultures and 1.36 to 1.43 for negative cultures (all *P* < .001) across PCT thresholds of 0.25, 0.50, 1.00, and 1.50 ng/mL (Supplementary Table 3).

## DISCUSSION

This large-scale study of 14 431 blood cultures from 2832 patients provides the first comprehensive quantification of the relationship between renal function and PCT values in patients with suspected bloodstream infections. We observed an inverse relationship between eGFR and positive PCT values in both culture-positive and culture-negative cases. For each successive category of decreased eGFR, the odds of PCT positivity were approximately 40% higher (OR 1.40 for positive cultures, 1.43 for negative cultures), after adjusting for age and race. As renal function declined, we observed higher proportions of positive PCT values—from 47.0% to 91.7% in culture-positive cases and 27.6% to 84.0% in culture-negative cases—suggesting that traditional PCT cutoff values become less reliable as renal function worsens. This effect was particularly pronounced in patients with eGFR <30 mL/min/1.73m², in whom PCT positivity occurred in more than 65% of all cases, regardless of culture status. The relationship between eGFR and PCT positivity was not affected by blood culture status.

Our sensitivity analyses across multiple PCT thresholds (0.25–1.50 ng/mL) demonstrated a consistent inverse relationship with renal function, providing further support for this association. Although previous studies have suggested using higher PCT thresholds in renal dysfunction, our findings raise fundamental questions about the diagnostic utility of PCT in this population.

These observations have implications for the utility of PCT as a diagnostic tool in patients with impaired renal function. For patients with normal renal function (eGFR ≥90 mL/min/1.73m²), PCT positivity rates showed a notable difference between positive and negative cultures (47.0% vs 27.6%, a difference of 19.4% points). However, this difference narrowed with declining renal function (91.7% vs 84.0%, a difference of only 7.7% points for eGFR <15 mL/min/1.73m [2]). This trend suggests that PCT's ability to distinguish between patients with and without bloodstream infections diminishes in the setting of severe renal impairment.

Our multivariate analysis revealed racial differences in PCT positivity that warrant further investigation. While we observed significantly higher odds of PCT positivity among Asian patients with positive cultures and Black/African American patients with negative cultures compared with White patients, these findings should be interpreted with caution given the predominantly White male veteran population in our cohort. In our extensive literature review, we found no previous studies examining racial differences in PCT operating characteristics, suggesting this may represent a novel observation. Future studies with more diverse populations are needed to validate these observations and explore potential biological or clinical factors contributing to racial differences in PCT values.

While PCT has been most extensively studied as a factor when determining the etiology (bacterial vs viral) and the duration of treatment for patients with LRTIs, we chose to study PCT in patients with suspected bacterial bloodstream infections for several reasons. First, unlike LRTIs where distinguishing between bacterial colonization and active infection can be challenging, blood cultures provide a definitive binary outcome (positive or negative), allowing for more reliable conclusions when assessing PCT's performance. Second, the FDA indication for PCT includes sepsis, a condition for which clinicians routinely order blood cultures as part of the diagnostic workup. When blood cultures are ordered, there is often clinical suspicion for sepsis or bloodstream infection. Despite this established clinical practice, the relationship between renal function and PCT values has been inadequately characterized specifically in patients with suspected bacteremia.

Our study has several limitations. The predominantly male, White, elderly veteran population limits generalizability to other patient groups, particularly to younger patients and women. Data were collected from a single institution. Our analysis captured renal function as a snapshot in time, without distinguishing between acute kidney injury, CKD, or acute-on-CKD. We lacked information about dialysis status, which may have a different effect on PCT values than non-dialysis-dependent renal dysfunction [25]. While we adjusted for age and race, unmeasured confounders such as inflammatory comorbidities, administered medications including antibiotics, and specific bacterial pathogens could influence the observed relationship between renal function and PCT values. Specifically, we lacked measures of illness severity, which may be higher in patients with CKD and could independently contribute to elevated PCT values. Additionally, our study focused specifically on suspected bloodstream infections, while PCT is commonly used in other contexts such as respiratory infections where this relationship may differ.

Future research should validate these findings in diverse populations and investigate whether the relationship between renal function and PCT varies by infection type or specific pathogens. Studies incorporating longitudinal measurements of both PCT and renal function could help characterize how dynamic changes in kidney function relate to PCT values over time. Additionally, research should quantify the relationship between PCT and eGFR in patients with acute kidney injury versus CKD, as well as in those undergoing various types of renal replacement therapy.

## Supplementary Material

ofaf654_Supplementary_Data

## Supplementary Data

Supplementary materials are available at *Open Forum Infectious Diseases* online. Consisting of data provided by the authors to benefit the reader, the posted materials are not copyedited and are the sole responsibility of the authors, so questions or comments should be addressed to the corresponding author.
