# Supplementary figures and images for: Quantifying the Relationship Between Renal Function and Procalcitonin: A Study of 14 431 Blood Cultures
Source: Open Forum Infect Dis. 2025 Oct 21;12(11):ofaf654. doi: 10.1093/ofid/ofaf654 (PMC12604010; doi:10.1093/ofid/ofaf654)

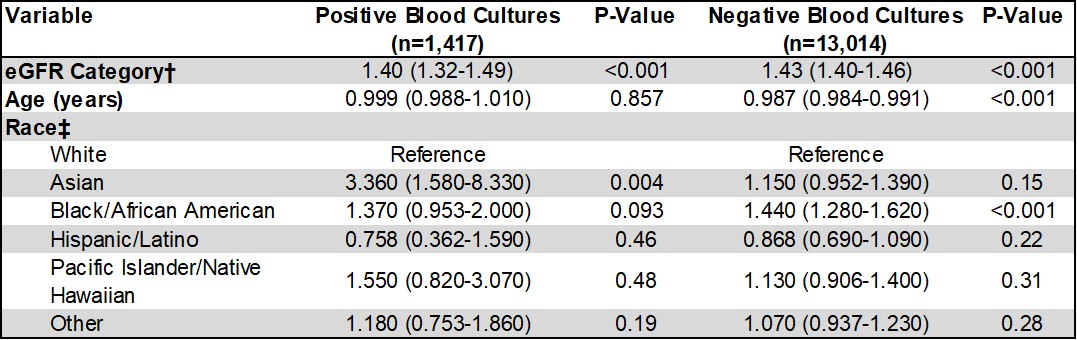

Supplement: ofaf654_Supplementary_Data [file ofaf654_supplementary_data.zip › Table S1.jpg]

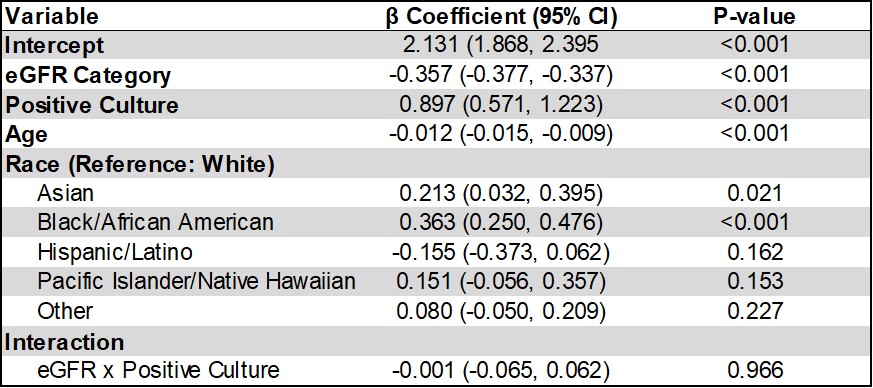

Supplement: ofaf654_Supplementary_Data [file ofaf654_supplementary_data.zip › Table S2.jpg]

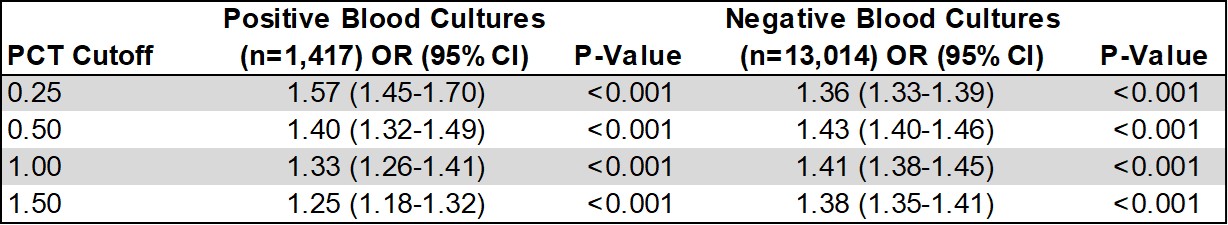

Supplement: ofaf654_Supplementary_Data [file ofaf654_supplementary_data.zip › Table S3.jpg]
